# Mesenchymal stromal cell transplantation ameliorates fibrosis and microRNA dysregulation in skeletal muscle ischemia

**DOI:** 10.1093/stmcls/sxae058

**Published:** 2024-09-16

**Authors:** Clara Sanz-Nogués, Alan J Keane, Michael Creane, Sean O Hynes, Xizhe Chen, Caomhán J Lyons, Emma Horan, Stephen J Elliman, Katarzyna Goljanek-Whysall, Timothy O’Brien

**Affiliations:** Regenerative Medicine Institute (REMEDI), University of Galway, Galway, Ireland; CÚRAM SFI Research Centre for Medical Devices, University of Galway, Galway, Ireland; Regenerative Medicine Institute (REMEDI), University of Galway, Galway, Ireland; Regenerative Medicine Institute (REMEDI), University of Galway, Galway, Ireland; Discipline of Pathology, University of Galway, Galway, Ireland; Division of Anatomic Pathology, University Hospital Galway, Galway, Ireland; Regenerative Medicine Institute (REMEDI), University of Galway, Galway, Ireland; Regenerative Medicine Institute (REMEDI), University of Galway, Galway, Ireland; Orbsen Therapeutics Ltd., Galway, Ireland; Orbsen Therapeutics Ltd., Galway, Ireland; Regenerative Medicine Institute (REMEDI), University of Galway, Galway, Ireland; Institute of Life Course and Medical Sciences, University of Liverpool, Liverpool, United Kingdom; Regenerative Medicine Institute (REMEDI), University of Galway, Galway, Ireland; CÚRAM SFI Research Centre for Medical Devices, University of Galway, Galway, Ireland

**Keywords:** mesenchymal stromal cells, peripheral arterial disease, muscle ischemia, microRNAs, fibrosis

## Abstract

Peripheral arterial disease (PAD) is associated with lower-extremity muscle wasting. Hallmark features of PAD-associated skeletal muscle pathology include loss of skeletal muscle mass, reduced strength and physical performance, increased inflammation, fibrosis, and adipocyte infiltration. At the molecular level, skeletal muscle ischemia has also been associated with gene and microRNA (miRNA) dysregulation. Mesenchymal stromal cells (MSCs) have been shown to enhance muscle regeneration and improve muscle function in various skeletal muscle injuries. This study aimed to evaluate the effects of intramuscularly delivered human umbilical cord-derived MSCs (hUC-MSCs) on skeletal muscle ischemia. Herein, we report an hUC-MSC-mediated amelioration of ischemia-induced skeletal muscle atrophy and function via enhancement of myofiber regeneration, reduction of tissue inflammation, adipocyte accumulation, and tissue fibrosis. These changes were observed in the absence of cell-mediated enhancement of blood flow recovery as measured by laser Doppler imaging. Furthermore, reduced tissue fibrosis in the hUC-MSC-treated group was associated with upregulation of miR-1, miR-133a, and miR-29b and downregulation of targeted pro-fibrotic genes such as Col1a1 and Fn1. Our results support the use of hUC-MSCs as a novel approach to reduce fibrosis and promote skeletal muscle regeneration after ischemic injury in patients with PAD.

## Graphical Abstract

**Graphical Abstract:**
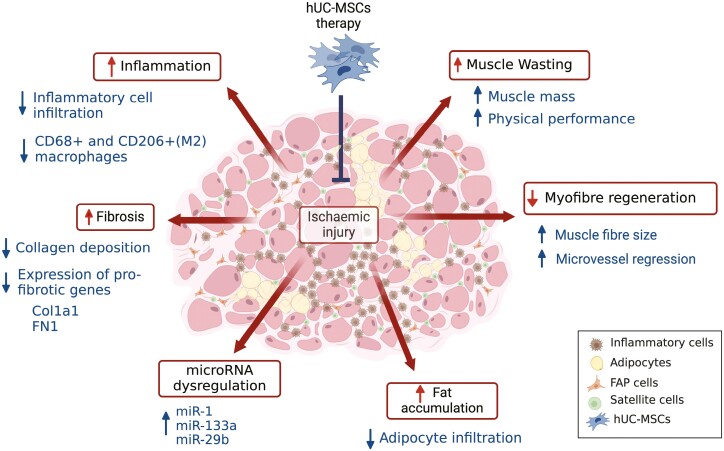

**Significance statement**

Ischemia results in pathophysiological changes in the muscle structure and function and an associated dysregulation of miRNA levels. Our results showed a significant reduction in inflammation, fibrosis, adipocyte infiltration, and an amelioration of muscle atrophy and dysfunction in ischemic muscles following umbilical cord-mesenchymal stromal cell (hUC-MSC) therapy. This was associated with partial restoration of miRNA levels (miR-1, miR-133a, and miR-29b) and a concomitant downregulation of their pro-fibrotic targets. These results suggest a potential role of hUC-MSCs in regulating ischemia-induced muscle fibrosis by modulating the levels of these miRNAs and their associated targets.

## Background

Peripheral arterial disease (PAD) is caused by hypoperfusion of the lower limb tissue (ie, ischemia) due to atherosclerotic disease.^1^ PAD can be asymptomatic or range from mild pain during physical activity (intermittent claudication (IC)) to critical limb-threatening ischemia (CLTI), a severe manifestation where pain occurs at rest, and gangrene and tissue loss may ensue.^1^ Although PAD is considered primarily a vascular disease characterized by reduced blood flow and altered angiogenesis/arteriogenesis, muscle wasting, ie, the loss of muscle mass, strength, and function, is often present in these patients.^2^ PAD-associated myopathy is characterized by reduced muscle mass, increased fat infiltration, fibrosis, inflammation, nerve dysfunction and impaired mitochondrial activity, oxidative stress, and peripheral nerve dysfunction.^2-7^ These pathophysiological changes can lead to a range of adverse outcomes including impaired balance, higher prevalence of falls, mobility loss, and a greater function impairment and decline,^8-10^ which place patients at higher risk of serious injury and hospitalization and increased risk of morbidity and mortality.^2,5,6,11^ Functional impairment and decline are common in PAD even in patients without obvious symptoms.^5^ At the molecular level, PAD has been associated with differential expression profiles of genes and microRNAs (miRNAs, miRs) in both the gastrocnemius muscle^12,13^ and circulation.^14-16^ miRNAs are small non-coding RNAs ~22 nucleotides in length which regulate gene expression at the post transcriptional level^17^ and are robust regulators of muscle development and homeostasis.^18^ In addition, they have been implicated in many diseases as pathological mediators, biomarkers, or therapeutic targets.^19^

Current therapeutic strategies for patients with PAD include exercise, lifestyle changes, and treatment of underlying risk factors, but these may only improve the clinical symptoms in mild cases.^1^ Lower limb revascularization surgery is the treatment of choice for CLTI.^20^ This enables blood flow restoration and limb salvage, while also reverse some muscle wasting features.^21^ Nevertheless, amputation rates are still high in these patients, approximately 15%-20% at 1 year, which is associated with higher morbidity and mortality risk (40% deaths at 1 year of amputation).^20^ Additionally, muscle wasting in patients with PAD has been correlated with more severe cases of artery stenosis.^22^ Thus, novel treatments that reverse the muscle wasting features in PAD may improve patients’ QOL and prevent disease progression.

Mesenchymal stromal cells (MSCs) have become an attractive cell source for treating various pathologies,^23^ including skeletal muscle diseases.^24^ MSCs’ secreted anti-apoptotic, anti-fibrotic, pro-angiogenic and matrix remodeling factors, as well as their immunomodulatory and anti-inflammatory properties are thought to be the primary mechanisms by which they promote endogenous tissue regeneration.^23^ MSC-mediated attenuation of muscle wasting has been previously attributed to an increased skeletal muscle mass and myofiber cross-sectional area, which results in increased muscle strength and endurance,^25^ or mediated by anti-apoptosis, anti-inflammatory, and mitochondrial biogenesis mechanisms.^26,27^ MSCs have also been shown to promote ischemic muscle repair by modulating the M1 to M2 macrophage switch.^28,29^

In this study, we investigated the therapeutic efficacy of human umbilical cord-derived mesenchymal stromal cells (hUC-MSCs) in treating ischemia-induced skeletal muscle damage in mice. Our results showed amelioration of ischemia-induced skeletal muscle inflammation, fibrosis, and muscle wasting features after hUC-MSC transplantation. We previously identified a miRNA dysregulation signature in ischemic muscles associated with fibrosis.^30^ Here, we investigated whether cell treatment restored the levels of these miRNAs and reduced fibrosis. Our results have shown, for the first time, the upregulation of miR-1, miR-133a, and miR-29b levels and a corresponding decrease in pro-fibrotic gene targets after hUC-MSC transplantation. Furthermore, we found a significant correlation between the levels of these miRNAs and features of muscle health.

## Methods

### Animals

Eight to 10 weeks old BALB/c nude male mice were purchased from Envigo (United Kingdom) and housed in a licensed preclinical facility at the University of Galway, with monitoring and support from qualified animal technicians and a veterinary surgeon. Ethical approval was obtained from the Institutional Animal Care Research Ethics Committee (ACREC). Project authorization was granted by the Health Products Regulatory Authority (HPRA) in Ireland.

### Cell culture

Human umbilical cord tissue was ethically sourced from Tissue Solutions Ltd. (Glasgow, UK). Anti-CD362+ selected hUC-MSCs were provided by Orbsen Therapeutics Ltd. Galway (Ireland). Primary isolation and expansion of anti-CD362+ selected hUC-MSCs were carried out as previously described.^31,32^ Briefly, a single-cell suspension was obtained from human umbilical cord tissue by enzymatic digestion and then stained with CD362-APC (clone 305515, dilution 1:50, R&D Systems). The CD362+ cells were then isolated using MS MACs column as per manufacturer’s instructions (Myltenyi Biotec). Cells were cultured in α-MEM containing 1% penicillin/streptomycin solution and supplemented with 10% fetal bovine serum (FBS) and 1 ng/mL basic fibroblast growth factor (b-FGF). Cells were kept at 37 °C, 5% CO_2_ and 2% O_2_. Cells were passaged using 0.05% trypsin-EDTA at∼80% to 90% confluence. Human UC-MSCs were used up to passage 3 for in vivo experiments.

### Induction of HLI

Unilateral HLI was surgically induced in nude mice as previously described by our group.^33^ Animals were anesthetized with 75 mg/kg ketamine and 0.5 mg/kg medetomidine (Domitor 10) solution injected subcutaneously. Anesthesia was partially reversed with atipamezole (5 mg/kg). The mice received analgesia (0.05-0.1 mg/kg of buprenorphine 8-12 h for 3 days, and as required thereafter) and prophylactic antibiotic (0.1 mg/kg of enrofloxacin/baytril) was also given once post-operatively. At 28 days after surgery, the animals were humanely euthanized, and their body weights were recorded.

### Laser Doppler imaging

A laser Doppler perfusion imager (MoorLDI V6.0, Moor Instruments, Axminster, UK) was used to assess foot perfusion. Blood flow was measured in the soles of both feet, before (Pre-) and immediately after HLI surgery (post-), on day 3 before cell injection, and on days 7, 14, 21, and 28 postsurgery. Anesthetized mice were placed on a surface at a constant temperature of 37 °C to reduce heat loss during the measurements. To account for ambient variables across time points, blood perfusion was expressed as the ratio of ischemic to non-ischemic limbs. The mean flux values were calculated using MoorLDIV6.0 image processing software.

### Intramuscular administration of cells

**Figure 1.**
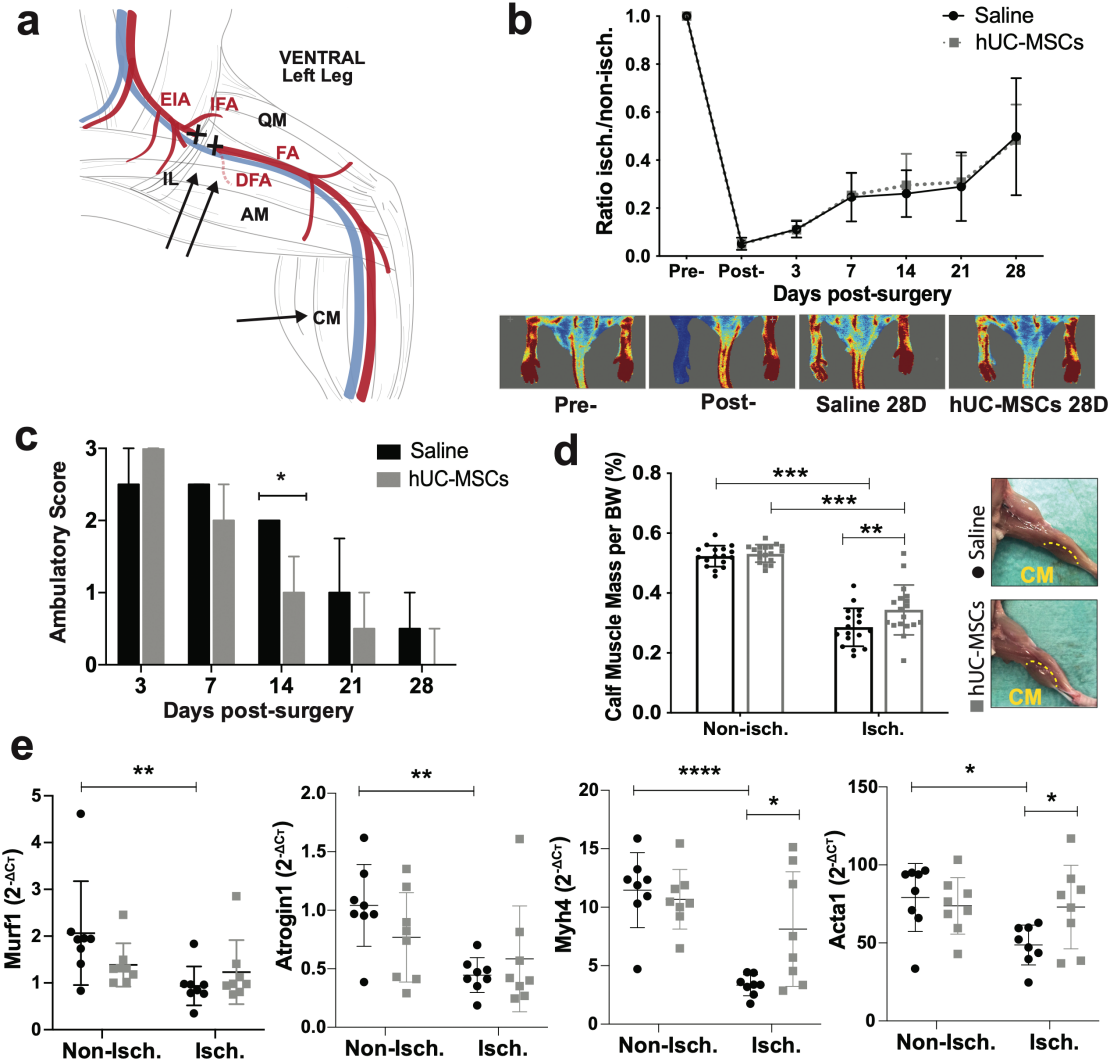
Transplantation of hUC-MSCs into BALB/c nude mice with HLI. a) Schematic representation of the cell injection sites (arrows) and ligation site (X symbol) in the HLI model. b) Blood flow perfusion of mouse foot using LDI over 28 days after surgery, *N* = 18 (hUC-MSCs) and *N* = 17 (Saline). Data are mean ± SD; 2-way ANOVA, Sidak’s multiple comparison test. c) Assessment of limb functionality using the ambulatory score (3 = dragging the foot; 2 = no dragging the foot but no plantar flexion; 1 = plantar flexion but no flexion of toes; 0 = flexion of toes to resist traction on the tail similar to the non-operated foot). Data are median ± IQR, *P < .05 (Mann-Whitney test). **d)** Percentage of calf muscle weight per body weight. Data are mean ± SD. ***P < .001; **P < .01 (2-way ANOVA, Sidak’s multiple comparisons test). d) mRNA expression levels of *Murf1*, *Atrogin1*, *Myh4*, and *Acta1* measured by RT-qPCR. *C*т values were normalized to *Rpl13a* using the 2^−ΔCт^ method. Data are mean ± SD; 2-way ANOVA, Sidak’s multiple comparison test (*N* = 8). BW = body weight; DFA = deep femoral artery; EIA = external iliac artery; IFA = iliacofemoral artery; FA = femoral artery; IL = inguinal ligament; QM = quadriceps muscle; AM = adductor muscle; CM = calf muscle.

A dose of 1 × 10^6^ hUC-MSCs was injected intramuscularly 3 days after HLI surgery. The cell pellet was resuspended in 90 μl of saline (sterile 0.9% NaCl). Mice received 3 injections of 30 μl per site: 2 injections were administered to the thigh muscles along the resected femoral artery, and one injection was administered to the calf muscles (Figure 1a). The control animals were administered saline under the same conditions. Randomization of treatments was performed using an allocation sequence unknown by the researchers’ performing injections and LDI analysis.

### Preclinical assessment of the ischemic limb

Limb functionality (ambulatory score) and the degree of ischemia (necrosis scores) were assessed on day 3 (before cell injection) and on days 7, 14, 21, and 28 postsurgery. The ambulatory score ranged from 0 to 3 depending on the limb mobility (3 = dragging the foot; 2 = no dragging the foot but no plantar flexion; 1 = plantar flexion but no flexion of the toes; 0 = flexion of the toes to resist traction on the tail similar to the non-operated foot) (see Supplementary Material S1). The necrosis scores ranged from 0 to 5 (5 = foot or limb amputation; 4 = severe necrosis affecting metatarsals; 3 = moderate cyanosis and/or mummification with or without amputation of phalanges; 2 = cyanosis or mild redness proximal and middle phalanges; 1 = mild cyanosis or redness of nails; 0 = normal).

### Tissue dissection and preparation

The calf muscles are the muscles most distant from the artery occlusion and are therefore expected to be the most affected by ischemic injury. For each animal, the posterior calf muscles from the ischemic and non-ischemic limbs were carefully dissected as previously reported,^34^ and the wet weight was recorded. Calf muscle mass was calculated as a percentage of body weight (calf muscle weight [g] × 100/body weight [g]). Tissue samples were fixed with 10% buffered formalin for 48 hours, processed using a Leica ASP3000 tissue processor and embedded in paraffin blocks.

### Histopathological assessment of ischemia severity

A semiquantitative scoring system was used to assess histopathological parameters of muscle ischemia, as previously described.^33^ Briefly, tissue cross-sections of 5 μm thickness were taken from the mid-belly of the dissected calf muscles. Four representative transverse sections across 2 different levels of the calf muscle were used for analysis. Tissue sections were rehydrated using a series of ethanol grades before staining with Haematoxylin and Eosin (H&E) and Mallory’s trichrome staining using standard protocols. Each parameter was independently scored by 3 blinded researchers using an analog scale ranging from 0 to 3. The presence of large extravascular accumulation of blood (hemorrhage) of unknown cause was observed focally in one hUC-MSC-treated muscle, therefore, an extra + 1 score was given to this mouse, according to our previous study.^33^ The cumulative ischemic severity score (cISS) was also obtained from the sum of each individual score.

### Immunohistochemistry

Mouse microcirculation was stained with 1:300 Dylight 594 conjugated Griffonia Simplicifolia Lectin I-isolectin B_4_ (GSL I-B_4_) (Vector Laboratory) for 1 hour. The number of microvessels per unit area (1/mm^2^) was quantified using the particle analyzer in ImageJ Version 2.0.0. Overall, a total of 12 representative pictures of sections across 2 different levels were taken with a 20× objective of an Olympus BX51 Upright Fluorescent Microscope and were quantified using the particle analyzer in ImageJ Version 2.0.0. Myofiber membranes were stained with 5 μg/mL fluorescein-conjugated wheat germ agglutinin (WGA; Vector Laboratories) for 10 minutes, and cell nuclei were counterstained with DAPI (Fluroshield, Sigma-Aldrich). The whole calf cross-section was scanned at 10× magnification using an EVOS M7000 Slide Scanner Microscope (Invitrogen). Quantitative determination of muscle fiber diameter (minimum Feret’s diameter) was performed using ImageJ Version 2.0.0. A total of 1500-2000 fibers per muscle were used for analysis. Tissue sections were stained with 1:100 of anti-CD68 (tissue macrophages, Abcam ab125212) and anti-CD206 (M2 activated macrophage-specific, Abcam ab64693) overnight at 4 °C, followed by 1:200 Alexa Fluor 488-conjugated secondary antibody (Abcam ab150077) according to the manufacturer’s protocol. Nuclei were counterstained with DAPI (Fluoroshield, Sigma-Aldrich). A total of 15 representative images across 2 levels were taken for analysis with the 4× objective of an Olympus BX51 Upright Fluorescent Microscope. The percentage of area fraction was calculated as the total percentage of green pixels in the image using ImageJ Version 2.0.0. Overall analysis was performed on injured/regenerating muscle zones.

### RNA isolation from paraffin-embedded sections

RNA was isolated from formalin-fixed paraffin-embedded (FFPE) calf skeletal muscle sections according to a protocol first described by Ma et al^35,36^ and further adapted by our group (see Supplementary Material S2).

### mRNA RT-qPCR

One thousand nanograms of total RNA were reverse transcribed using the Invitrogen Superscript IV system according to the manufacturer’s instructions. qPCR was performed in 10 μL reactions using Applied Biosystems Fast SYBR Green Master Mix on an Applied Biosystems StepOnePlu Real-Time PCR System. Samples were run in technical duplicates and a no-template control (NTC) was run for each primer pair using molecular grade H_2_O instead of cDNA. A primer concentration of 200-500 nM and annealing temperature of 60-62 °C were used. The relative expression of target mRNAs was normalized to Rpl13a expression using the 2^-ΔCт^ method. Experimentally validated primer sequences were retrieved from PrimerBank.^37^ Primer sequences are shown in Supplementary Material S3. The aggregate expression of collagen type I alpha 1 (*Col1a1*), fibrin 1 (*Fn1*), and alpha 2 smooth muscle actin (*α-SMA,* also known as *Acta2*). *Acta2* was used to give a “fibrotic score” as previously described by Arrighi et al.^38^ Briefly, for each marker, the normalized expression value for each sample was divided by the maximum expression value (Exp Max) of that marker across all samples. The average value of the 3 targets gave a fibrotic score (FS) between 0 and 1 for each sample which was calculated using the following formula:


$$\begin{array}{l} FS=\frac{1}{3}\left[\frac{Exp(COL1A1)}{ExpMax(COL1A1)}+\frac{Exp(FN1)}{ExpMax(FN1)}+\frac{Exp(ACTA2)}{ExpMax(ACTA2)}\right] \end{array}$$

### miRNA RT-qPCR

To assess skeletal muscle miRNA expression, 100 ng of total RNA was reverse transcribed using the Qiagen miRCURY LNA RT kit according to the manufacturer’s protocol. cDNA was diluted 1:60 and subsequently used in RT-qPCR reactions using the miRCURY LNA qPCR SYBR Green kit and primer assays (Supplementary Material_S4) on an Applied Biosystems StepOnePlus Real-Time PCR System.

### Statistical analysis

The researchers responsible for processing the samples and analyzing the data during the study were blinded, throughout to minimize bias in the analysis. Overall, results are expressed as mean ± SD for continuous variables or median ± interquartile range (IQR) for ordinal variables. The D’Agostino & Pearson test was used to determine whether the sample data came from a normally distributed population. The differences between the 2 groups were tested using an unpaired *t* test (for normally distributed data) or the nonparametric Mann-Whitney *U* test. Differences among multiple groups were tested statistically using 1- or 2-way analysis of variance (ANOVA) with post hoc testing. Pearson’s or Spearman rank-order correlation was performed between muscle parameters, depending on whether they were continuous or ordinal variables, respectively. All statistical analyses were performed using GraphPad Prism Version 9 statistical software. Statistical significance was set at *P* ≤ .05.

## Results

### Transplantation of hUC-MSCs improved skeletal muscle atrophy and function but did not enhance blood flow perfusion

HLI surgery immediately reduced the blood flow perfusion to 5% in both groups (Figure 1b). Three days after HLI surgery, cells and saline treatments were delivered intramuscularly (Figure 1a). Overall, there was no statistically significant difference between the LDI measurements on day 3, before cell/vehicle administration (0.11 ± 0.03 in the saline-treated group and 0.11 ± 0.04 in the hUC-MSCs-treated group), indicating that the randomization process was successful. Blood flow recovery increased over time but was still impaired on day 28. No significant differences were observed between the 2 treatment groups at any time point (Figure 1b). Along with the initial postsurgery drop in blood flow, mice displayed reduced walking ability which was restored over time in both treatment groups. Nevertheless, hUC-MSC-treated mice showed superior leg/foot mobility compared with saline-treated mice, which was statistically significant at day 14 post-HLI (Figure 1c). Necrosis was observed only in the nails and/or top of phalanges in 2/18 hUC-MSC-treated mice and 3/17 saline-treated mice. Twenty-eight days of persistent ischemia resulted reduced calf muscle mass. However, a statistically significant increase of overall calf muscle mass percentage per body weight was observed in the muscles treated with hUC-MSCs compared to those treated with saline (*P* = .008). Muscle atrophy in saline-treated mice was also apparent on gross examination (Figure 1d). We analyzed markers of muscle atrophy which are regulators of muscle mass, including the muscle-specific ubiquitin ligases muscle RING-finger protein-1 (*Murf1*) (also known as *Trim63*) and *Atrogin1* (also known as *Fbxo32*)^39,40^ (Figure 1e). *Murf1* and *Atrogin1* expression was significantly suppressed in ischemic limbs of saline-treated mice while there was no significant hUC-MSC effect observed. We also analyzed the expression levels of muscle myosin heavy chain 4 (*Myh4*), which is largely expressed in mouse skeletal muscle,^41^ and alpha skeletal muscle actin (*Acta1*). These 2 genes are related to skeletal muscle phenotype and structure.^42,43^ Our results showed reduced expression of *Myh4* and *Acta1* in the ischemic limb of saline-treated mice vs the contralateral non-ischemic limb, and a significant upregulation in the expression of these 2 genes in hUC-MSC-treated muscles (Figure 1e).

### hUC-MSC transplantation ameliorated ischemia-induced skeletal muscle damage

**Figure 2.**
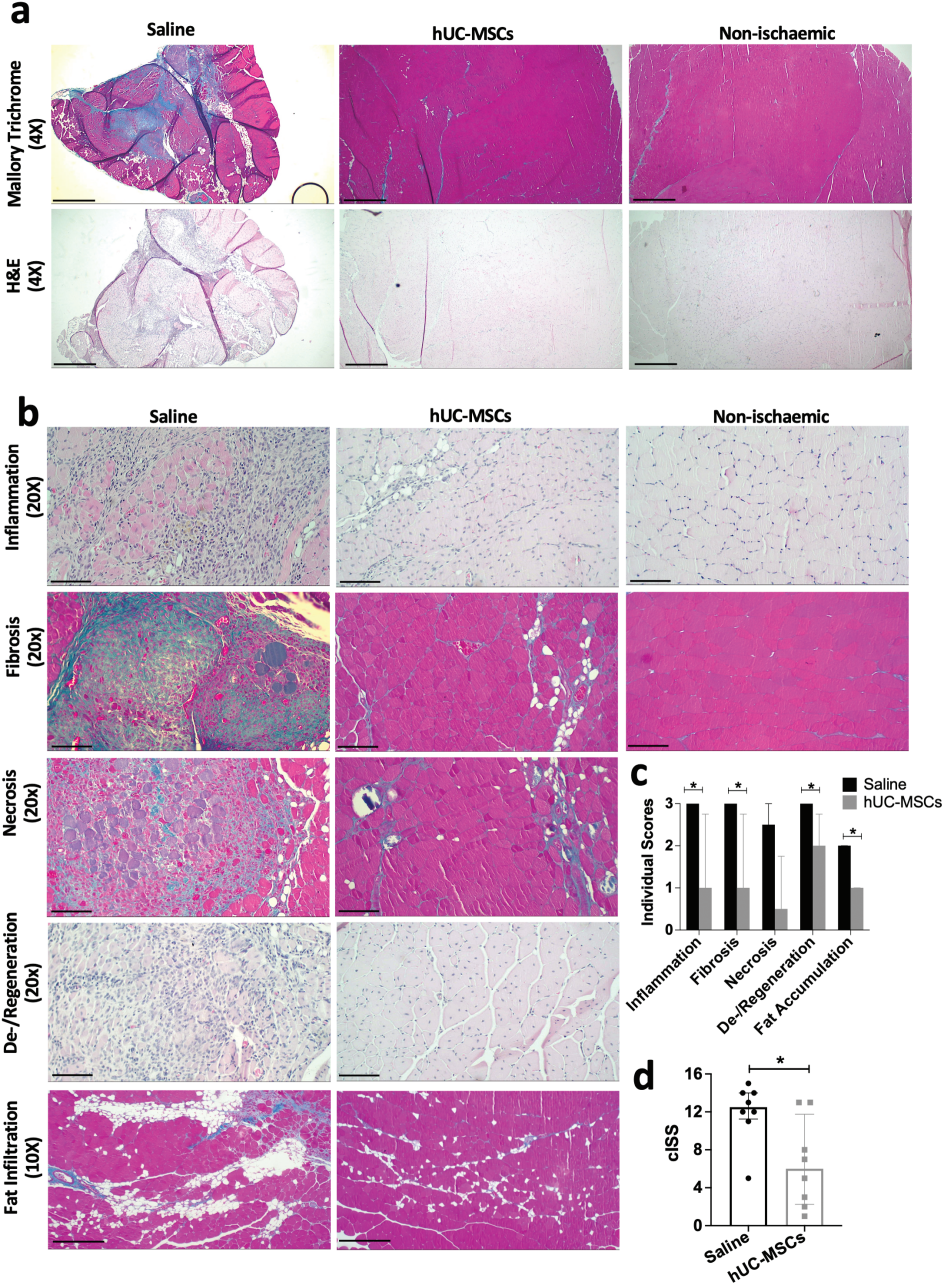
Semi-quantitative histopathological assessment of ischemic damage in calf skeletal muscle. a) Representative images of skeletal muscle sections stained with Mallory’s trichrome and H&E. Scale bar: 4X = 250 μm. b) Representative images of the individual skeletal muscle parameters scored. c) Individual ischemia severity scores. d) Cumulative ischemia severity score (cISS). Data are median ± IQR. *N* = 8 saline, *N* = 8 hUC-MSCs; *P < .05 (Mann-Whitney test). Scale bar 10× = 250 μm; 20× = 50 μm.

The overall extent of muscle ischemia-induced damage was quantified using a semiquantitative scoring system.^33^ Skeletal muscle treated with saline showed significant muscle atrophy with reduced overall cross-sectional calf area by gross examination, compared to muscle treated with cells (Figure 2a). Several histopathological parameters were evaluated independently (Figure 2b). *Inflammation*: calf muscle treated with saline presented severe inflammation characterized by an accumulation of leukocytes scattered across large areas or present focally in large clusters, accompanied by a significant loss of muscle fiber integrity and other morphological changes such as necrosis and fibrosis. Most hUC-MSC-treated muscles had normal appearance or mild inflammation with no significant damage to muscle fibers, and 3/8 mice had moderate to severe inflammation. *Fibrosis*: there was a marked presence of intermuscular collagen fibrillar material (blue in Mallory Trichrome staining) with a generalized loss of tissue architecture affecting several myofiber bundles in saline-treated muscles, and a significant reduction in the level of fibrosis in the hUC-MSC-treated muscles. *Necrosis*: Muscles from saline-treated mice had large clusters of necrotic fibers stained purple under Mallory’s trichrome stain, along with severe inflammation and fibrosis. Most of the muscles treated with cells had no necrosis or a mild version of necrosis characterized by dystrophic mineralization or calcification affecting only a few single fibers. *Fiber degeneration*: Saline-treated muscles showed predominant degeneration of myo fibers characterized by the presence of large areas of marked attenuation or disruption of several muscle fiber bundles accompanied by the presence of severe inflammation and fibrosis. In the hUC-MSC-treated muscles, there were large areas of markedly more homogeneous, small-to-medium-sized myofibers, with centralized nuclei present, which is a signature of skeletal muscle regeneration. *Adipocyte infiltration*: Large clusters or widely spread inter-muscle adipocyte infiltration was observed in most saline-treated mice, while normal levels or mildly scattered inter-muscle accumulation of adipose tissue was observed in the hUC-MSC-treated mice. The final total severity score was calculated for each animal, by adding up the individual scores (Figure 2c). Treatment with hUC-MSCs resulted in an overall amelioration of ischemia-induced tissue damage (cISS = 6, mild ischemic damage) compared with saline-treated mice (cISS = 12.5, severe ischemic damage; Figure 2d).

### hUC-MSC transplantation supported macrophage clearance in ischemic muscle

**Figure 3.**
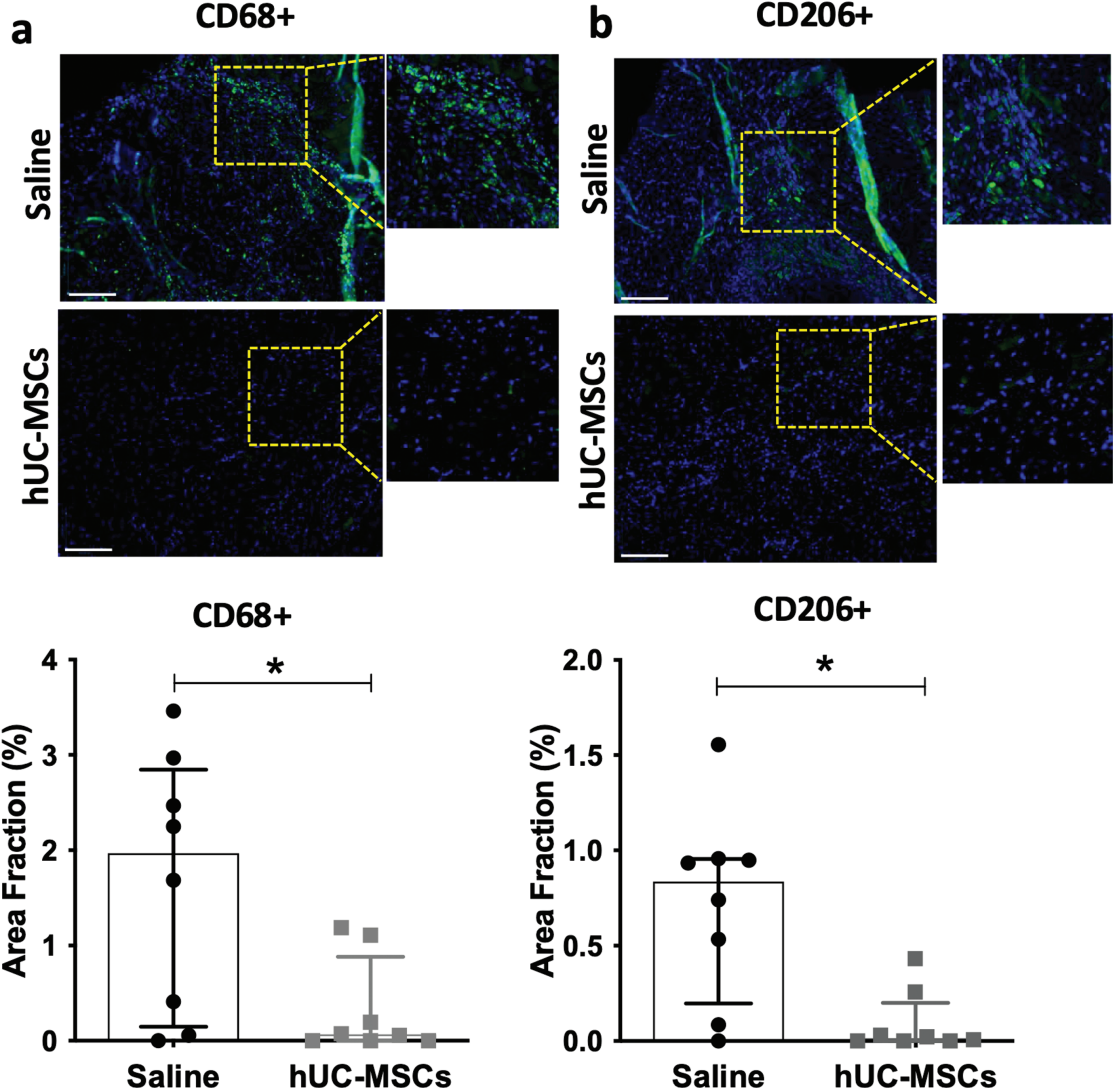
Effect of hUC-MSC transplantation on skeletal muscle inflammation. Representative immunostaining and quantification of CD68+ cells (tissue macrophages) (**a**) and CD206+ cells (M2 macrophages) (b) Nuclei were counterstained with DAPI. Scale bar = 130 μm. Data are median ± IQR; *N* = 8 mice per group; *P-value < .05; (Mann-Whitney test).

To investigate the potential modulatory effect of hUC-MSC transplantation on local inflammation, we examined the levels of macrophages present in ischemic tissue. Cells expressing the tissue macrophage-specific antigen CD68 were still present in ischemic sites 28 days after surgery. The number of CD68+ macrophages was significantly higher in the control group than in the hUC-MSCs-treated group (*P* = .044) (Figure 3a). Similarly, macrophages expressing the alternatively activated M2 macrophage-specific antigen CD206 were also present in ischemic foci on day 28 post-surgery in the saline-treated group, but not in the hUC-MSC-treated group (*P* = .0107; Figure 3b).

### hUC-MSC transplantation enhanced muscle regeneration after ischemic injury

**Figure 4.**
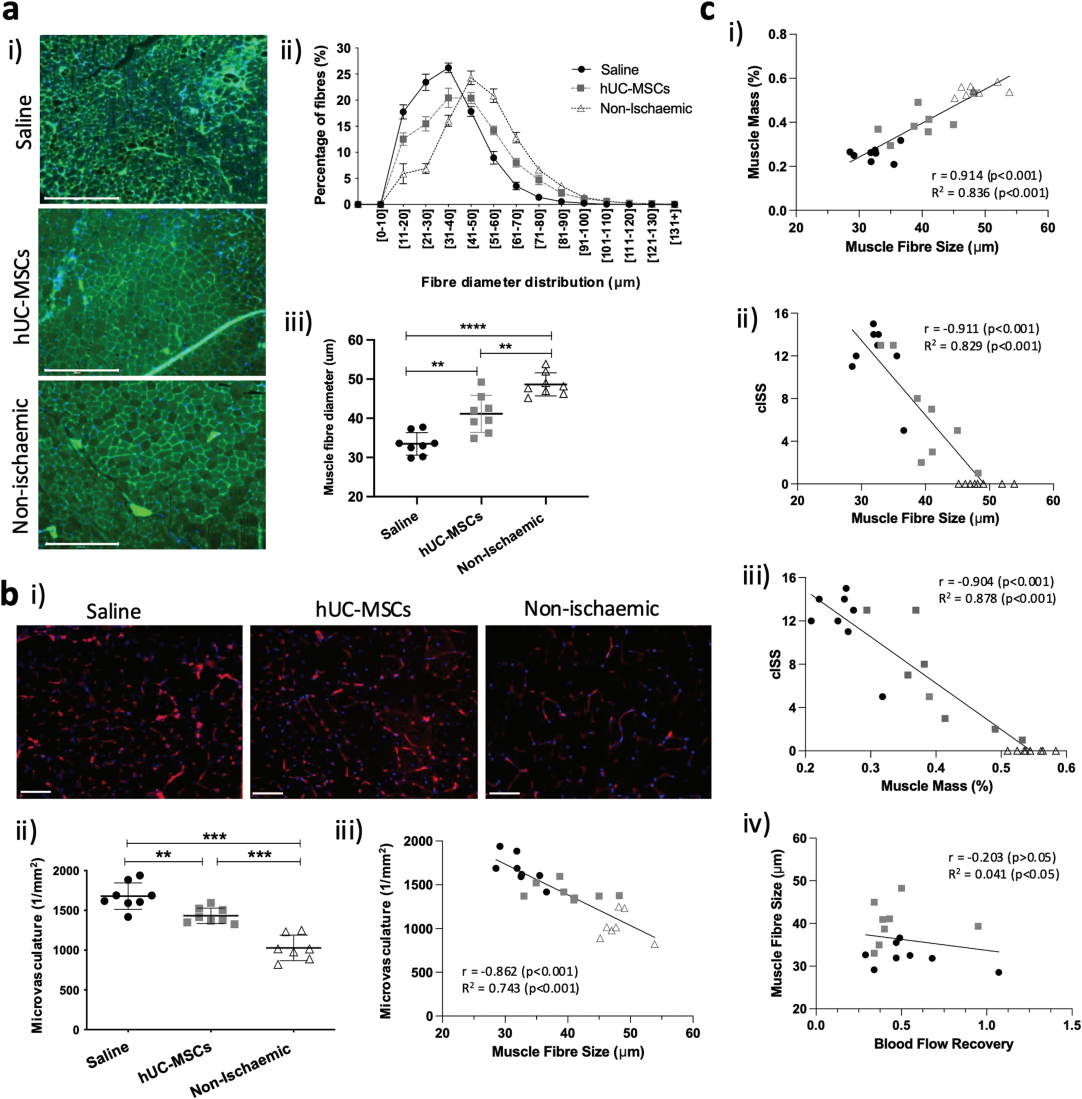
Effects of hUC-MSC transplantation on skeletal muscle regeneration after ischemic injury. a-i) Fluorescence staining of skeletal muscle cross-sections labeled with WGA and nuclei counterstained with DAPI. *b*. Scale bar = 275 μm. a-ii) Quantitative determination of skeletal muscle fiber size distribution (minimal Feret’s diameter). Data are mean ± SEM. a-iii) Median values of total myofibre diameter (mean ± SD) **P < .01 ***P < .001 (one-way ANOVA, Tukey’s multiple comparison test). b-i) Representative images of the mouse microvasculature stained with Isolectin GS-IB4 and nuclei counterstained with DAPI. Scale bar = 58 μm. b-ii) Quantification of mouse microvasculature/mm^2^. Data are mean ± SD); **P < .01; ***P < .001 (one-way ANOVA, Tukey’s multiple comparison test). b-iii) Correlation and linear regression analyses between microvasculature density, cumulative ischemic severity score (cSSI) and muscle fiber diameter. c-i-iv) Correlation and linear regression analysis between % calf muscle mass, cumulative ischemic severity score, muscle fiber size, and blood flow perfusion.

Skeletal muscle samples from hUC-MSC-treated mice presented small-to-medium-sized myofibers with centralized nuclei by H&E staining. Saline-treated muscles had large areas of attenuated or disrupted muscle fibers (Figure 2b). Skeletal muscle size distribution showed a higher percentage of smaller fibers in saline-treated mice compared with cell-treated group and non-ischemic muscle (Figure 4a), with a median myofiber size of 33.5 ± 2.9, 41.1 ± 4.7, and 48.6 ± 2.9 μm diameter, respectively. The enlargement of myofibers in the hUC-MSC-treated muscle was accompanied by a reduction in the total number of microcirculation vessels per unit area, which was significantly higher in the saline-treated group (1679 ± 167 vessels/mm^2^) than that in the hUC-MSC-treated group (1432 ± 97 vessels/mm^2^) and non-ischemic muscles (1028 ± 161 vessels/mm^2^; Figure 4b). Our analysis showed a statistically significant negative correlation between the number of microcirculation vessels and muscle fiber size (*r* = –0.87, *P* < .001; Figure 4c-i). In addition, there was a strong positive correlation between myofiber size and % calf muscle mass (*r* = 0.91, *P* < .001) (Figure 4c-ii), which was negatively correlated with the level of ischemic damage determined by the cISS (*r* = –0.90, *P* < .001; Figure 4c-iii). We found no significant association between blood flow perfusion measured by LDI and any of the muscle parameters analyzed (Figure 4c-iv).

### hUC-MSC transplantation reduced skeletal muscle fibrosis

**Figure 5.**
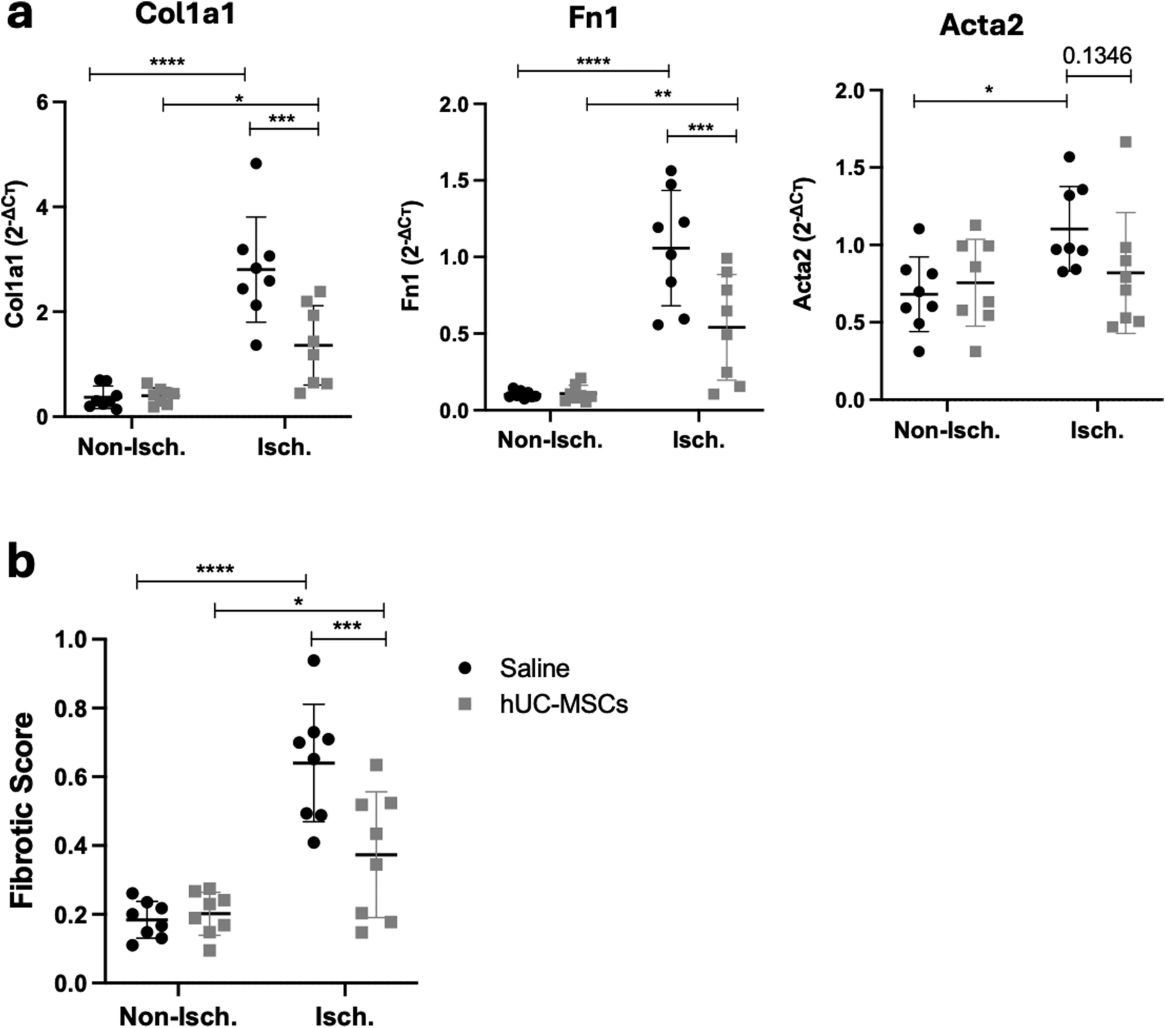
Transplantation of hUC-MSCs regulates skeletal muscle fibrosis. a) mRNA expression levels of *Col1a1*, *Fn1*, and *Acta2* measured by RT-qPCR. *C*т values were normalized to *Rpl13a* using the 2^−ΔCт^ method. **b)** Fibrotic score calculated as the aggregated expression value of the 3 pro-fibrotic markers by RT-qPCR (range 0-1). Data are mean ± SD, *P < .05, **P < .01, ***P < .001 (two-way ANOVA, Sidak’s multiple comparison test).

Interstitial collagen deposition is often accompanied by impaired muscle regeneration after injury. Our histological observations indicated increased fibrosis in saline-treated mice and an amelioration of fibrosis after cell treatment. We used RT-qPCR to measure the expression of pro-fibrotic genes such as *Col1a1*, *Fn1*, and *Acta2*. Results showed a significant upregulation of these 3 pro-fibrotic markers 28 days after ischemic injury, and a significant downregulation of *Col1a1* and *Fn1* expression in hUC-MSC-treated muscle compared with saline-treated muscle (Figure 5a). A fibrotic score based on the normalized expression of *Col1a1*, *Fn1*, and *Acta2* was calculated to evaluate the extent of fibrosis in ischemic muscle samples, as previously described.^38^ In the work from Arrighi et al, the authors classified the extent of fibrosis in muscle biopsies as “low” when the score was equivalent to that of control biopsies (< 0.25) and “high” for those samples with fibrotic score > 0.25. Similar to the results of Arrighi et al results, the baseline fibrotic score of non-ischemic samples was <0.25 (median 0.197, IQR [0.138, 0.2415]) therefore considered as “low.” Most of the ischemic muscle samples had a ‘high’ fibrotic score of >0.25. However, results showed a statistically significant reduction in the overall fibrotic score in hUC-MSC-treated mice (median 0.440, IQR [0.197, 0.542]) compared with saline-treated mice (median 0.679, IQR [0.544, 0.88]), which agreed with our histological observations (Figure 5).

### hUC-MSC transplantation restored miRNA dysregulation associated with ischemic muscle damage

**Figure 6.**
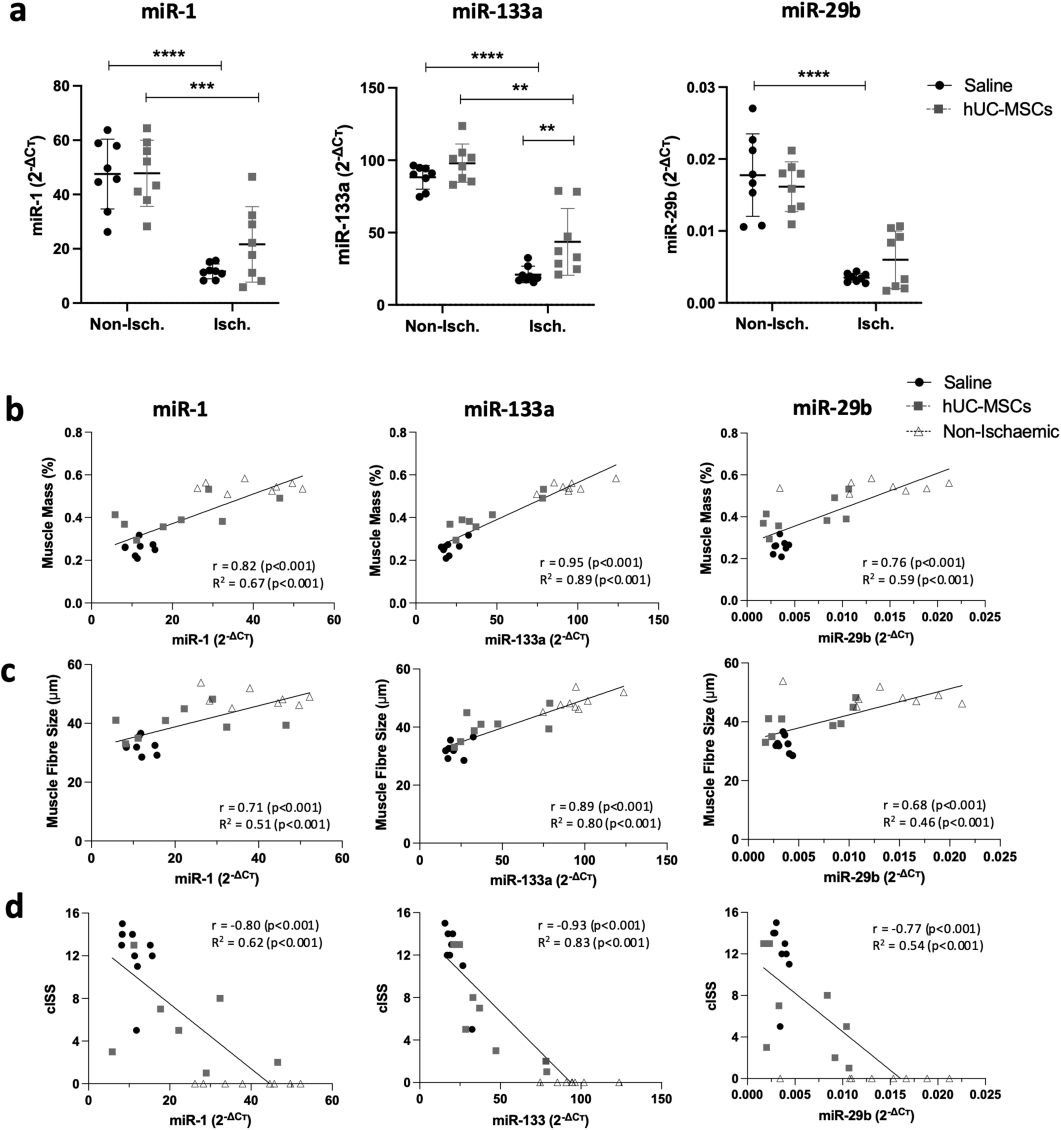
miR-1, miR-133a, and miR-29b expression levels correlated with skeletal muscle health parameters. a) Expression levels of miR-1, miR-133a, and miR-29b measured by RT-qPCR. *C*т values were normalized to Snord68 using the 2^−ΔCт^ method. Correlation analysis of miRNA levels with % muscle mass (b), muscle fiber size (c), and cumulative ischemic severity score (cISS) (d). Data are mean ± SD; **P < .01; ***P < .001 (2-way ANOVA, Sidak’s multiple comparison test).

Previous work from our group identified several miRNAs including miR-1, miR-133a, and miR-29b dysregulated in human CLTI muscle. The downregulation of these miRNAs was confirmed in ischemic mouse muscle 7 days post-ischemic injury.^30^ In this study, we have investigated the effect of ischemia and cell delivery on the expression levels of these miRNAs at 28 days post-ischemia. Our results showed that miR-133a, miR-1, and miR-29b were significantly downregulated at day 28 post-HLI in skeletal muscle and that the miRNAs levels were partially restored after cell treatment. Although only miR-133a reached statistical significance (Figure 6a), there was a noticeable trend toward increased levels of miR-29b and miR-1 in hUC-MSC-treated muscle. In addition, we found that the levels of these 3 miRNAs were significantly associated with muscle health parameters including a gain in muscle mass (Figure 6b) and muscle fiber diameter (Figure 6c). A statistically significant negative correlation between these 3 miRNAs and the level of ischemia severity (represented as cISS) was also observed (Figure 6d).

## Discussion

**Figure 7.**
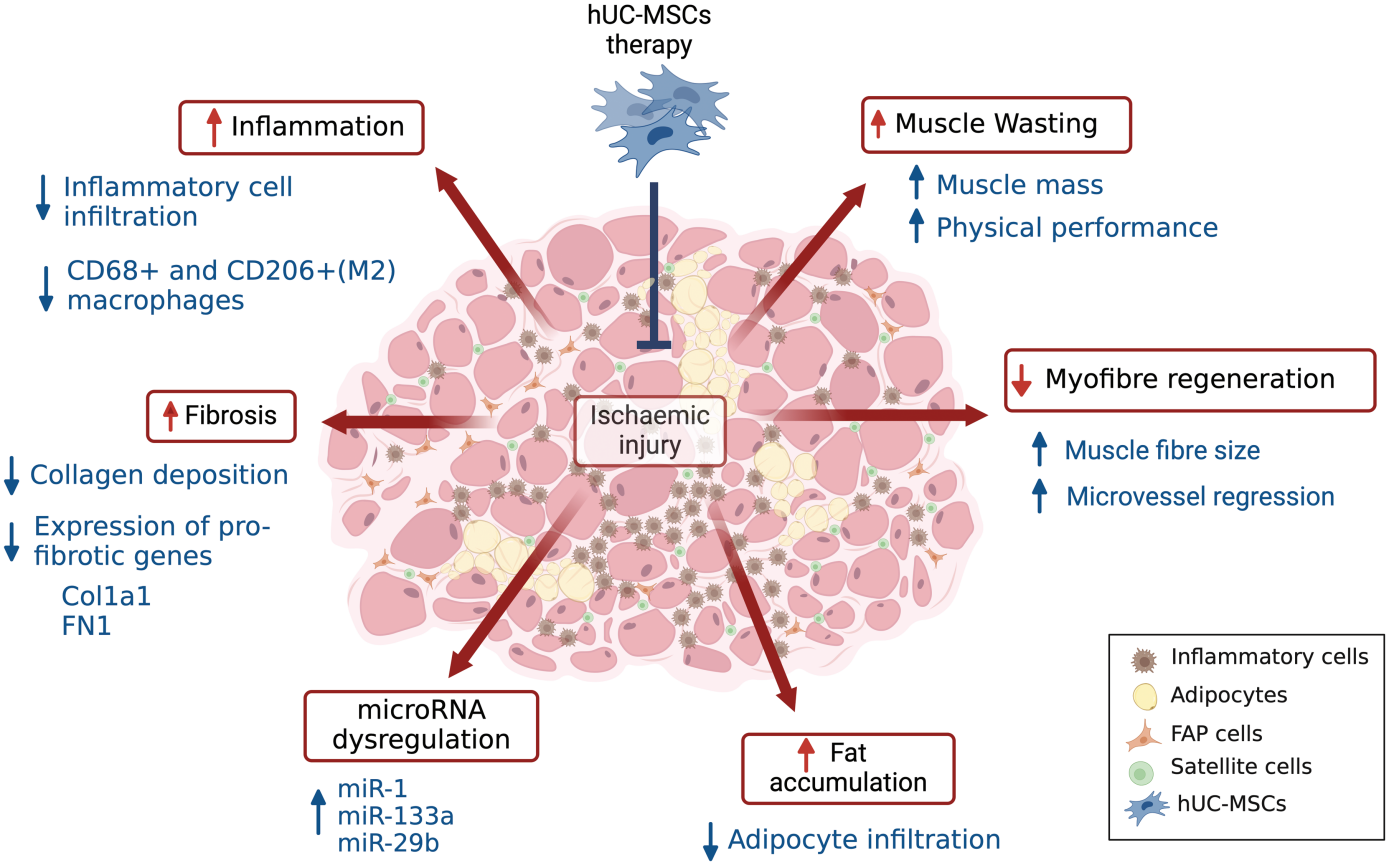
Schematic representation of the pleiotropic effects of hUC-MSC on skeletal muscle damage after sustained ischemic injury. Ischemic injury results in muscle mass loss and dysfunction and a variety of skeletal muscle abnormalities such as increased inflammation, fibrosis, fat accumulation and microRNA dysregulation, which results in impaired myofiber regeneration and enhanced muscle atrophy. Transplantation of hUC-MSCs into ischemic skeletal muscle restored muscle mass and function, enhanced skeletal muscle regeneration and repair, partially restored the levels of dysregulated miRNAs, and reduced the expression levels of associated pro-fibrotic genes. Created in BioRender (BioRender.com/d48h782).

In this study, we examined the therapeutic potential of hUC-MSCs in treating ischemic skeletal muscle damage. The principal findings of our study are as follows: 1) cell treatment enhanced calf muscle mass and improved physical performance in mice compared to saline; 2) this was accompanied by enhanced myofiber regeneration in hUC-MSC-treated mice, as seen by the enlargement of myofiber diameter, the presence of centralized nuclei, and regression of the microvascular network toward the levels of non-ischemic muscle^44^; 3) cell treatment resulted in a reduction of overall skeletal muscle inflammatory cell infiltration, including reduced levels of CD68 + and CD206 + macrophages; 4) hUC-MSCs resulted in a reduction of adipocyte and collagen fibrillar intermuscular deposition and downregulation of expression of pro-fibrotic genes such as *Col1a1* and *Fn1*; 5) cell treatment resulted in an upregulation of miR-1, miR-133a, and miR-29b, which are known to target these pro-fibrotic genes (see Supplementary Material_S5) The pleiotropic effects of hUC-MSCs on ischemia-induced skeletal muscle atrophy and dysfunction are summarized in Figure 7.

The mechanisms of skeletal muscle regeneration after injury include a complex and well-coordinated temporal sequence of cellular and molecular events that involves a variety of cell players from the muscle niche, including myogenic progenitor cells (MPCs; also known as satellite cells), myofibers, endothelial cells, dermal fibroblasts, fibro-adipogenic progenitors (FAPs), and inflammatory cells.^45,46^ Among all these different cell types, macrophages have emerged as the central players in coordinating different cellular and molecular processes associated with skeletal muscle regeneration and repair.^47,48^ After muscle injury, two distinct subpopulations of macrophages have been shown to sequentially invade the injured tissue: pro-inflammatory M1 macrophages, responsible for phagocytosis and removal of necrotic debris, and anti-inflammatory phenotype (M2), responsible for the resolution of inflammation and regeneration of myofibres.^49^ The transition of macrophages from M1 to M2 phenotype is responsible for coordinating the processes of angiogenesis, inflammation, myogenesis, and tissue remodeling.^45^ Having an appropriate spatiotemporal balance of macrophage phenotypes is essential for establishing an inflammatory microenvironment that is favorable for skeletal muscle regeneration and returning to tissue homeostasis.^47,48^ On the other hand, dysregulation of macrophage accumulation or polarization due to trauma, disease or age has been shown to result in impaired muscle regeneration and pathologic fibrosis.^47,50^ Our histological analysis of muscle at day 28 post-ischemic injury showed a persistent infiltration of inflammatory cells, including CD68+ and CD206+ macrophages, as well as a significant increase in tissue fibrosis in saline-treated muscles compared to those treated with hUC-MSCs. The M2-macrophage-specific marker CD206 is expressed in the M2a and M2c phenotypes.^51^ While these subsets of macrophages have been shown to contribute to tissue repair and angiogenesis, their persistent accumulation in injured tissue has been associated with unfavorable pro-fibrotic effects and tissue scar formation, especially during later stages of tissue repair.^50,52^ This may explain the enhanced levels of tissue fibrosis observed in the saline-treated group. In addition, our findings showed an increase in intermuscular fat accumulation which was significantly reduced by hUC-MSC treatment. FAPs are a population of muscle-resident MSCs with the potential to differentiate into adipocytes and fibroblasts and play a critical role in the maintenance of muscle homeostasis and regeneration.^53-55^ Dysregulation of the adipogenic and fibrotic differentiation of FAPs due to age, trauma or disease can lead to abnormal intermuscular fat infiltration and excessive fibrosis resulting in muscle loss and dysfunction.^56^ Thus, FAPs have recently been proposed as key cells in tuning skeletal muscle regeneration from compensatory to pathogenic.^53^ Furthermore, Abbas et al recently showed that the genetic ablation of Pax7 + satellite cells in a Pax7 deficient (Pax7^Δ^) mouse model resulted in the complete absence of muscle regeneration and a significant FAP-mediated increased in adipocyte accumulation that replaced functional muscle and reduced muscle force.^57^ This study confirmed the critical function of Pax7+ satellite cells in muscle regeneration following ischemic injury.

Our results showed that hUC-MSC treatment resulted in an increased calf muscle mass and enhanced walking ability. *Atrogin1* and *Murph1*, which are common markers of muscle atrophy, were downregulated in the ischemic limb, a finding also reported at 7 days post-HLI.^30,58^ Although no significant hUC-MSC-mediated change in *Murf1* and *Atrogin1* expression was found, there was a significant increase of muscle-mass related genes *Myh4* and *Acta1* after hUCM-MSC treatment. Previous studies have shown that knockdown of *Acta1* and *Myh4* in mice result in severe skeletal muscle atrophy and premature postnatal death.^59,60^ These 2 genes encode myosin heavy chain-IIB (MyH-IIB) and skeletal muscle α-actin, respectively, which are 2 fundamental components of the sarcomere responsible for force production, muscle contraction, and movement.^42,43^ The increased expression of *Acta1* and *Myh4* is in concordance with better ambulatory capacity observed in mice treated with hUC-MSCs. The reduced inflammation, fibrosis, and fat accumulation observed in muscles treated with hUC-MSCs, together with the enlarged myofiber size is likely to have contributed to this observation too.^49^ As muscle fiber size increased, we observed a decrease in the number of microvessels toward baseline levels. Interestingly, saline-treated muscles had enhanced microvascular density compared with hUC-MSCs and non-ischemic muscles. Although physiological angiogenesis is necessary for skeletal muscle regeneration, abnormal or pathological vascular proliferation may not result in enhanced muscle regeneration. For instance, White et al reported a higher number of capillaries in muscles from people with PAD than in those without PAD, which was not associated with better functional performance.^61^ The increased capillary density observed in patients with PAD may represent a compensatory mechanism for ischemia; however, this phenomenon may not be sufficient to overcome the adverse effects of muscle ischemia.^7^ Ochoa et al have previously described a working model in wild-type mice which display the temporal sequence of cellular and molecular events of inflammation, angiogenesis, and skeletal muscle regeneration following injury.^44^ We observed a strong correlation between an increase in myofiber diameter and muscle mass, and a decrease in microvasculature density, which is supported by the findings of Ochoa et al. A physiological explanation for this observation is that with the progressive increase in regenerated muscle fibers, the metabolic demands of the growing myofibers decrease and thus the expression of anti-angiogenic factors follows, contributing to the regression of capillaries toward baseline levels.^44^ Our histological results, therefore, indicate that at day 28 post-ischemia, hUC-MSC-treated muscles were in a more advanced stage of muscle regeneration, characterized by increased muscle fiber size and muscle mass, and regression of microvasculature density toward baseline levels. Saline-treated muscles showed impaired regeneration which remained in a pro-inflammatory and pro-fibrotic stage at day 28 post-ischemia with increased adipocyte infiltration. Interestingly, hUC-MSC-mediated changes in the skeletal muscles were observed even in the absence of an enhancement of tissue blood perfusion (eg, by enhanced collateral formation). Contrary to our observations, other studies have shown the ability of MSCs to enhance blood flow recovery.^28,29,62-64^ This suggests that other skeletal muscle cell-specific mechanisms independent of blood supply contribute to skeletal muscle homeostasis after ischemia. We hypothesize that exogenous delivery of hUC-MSCs may directly or indirectly interact with key cellular players in the ischemic skeletal muscle niche, including macrophages, satellite cell and FAPs to resolve inflammation, enhance regeneration and reparative processes, and restore the muscle microenvironment after ischemia. It is likely that MSCs exert their regenerative capacity through paracrine effects and immunomodulation, *via* secretion of growth factors, chemokines, cytokines and other biologically active molecules (eg, miRNAs) either as soluble molecules or enclosed in extracellular vesicles. Recent studies have focused on elucidating the mechanism of MSC-mediated polarization of macrophages into M2 alternatively activated phenotypes, identifying prostaglandin E2 (PGE-2),^65^ tumor necrosis factor-stimulated gene and protein 6 (TSG-6),^66^ indoleamine-pyrrole 2,3-dioxygenase (IDO),^67^ and the uptake of MSC mitochondria-containing extracellular vesicles^68^ or MSC apoptosis-mediated immunomodulation^69^ as potential mechanisms for MSC-mediated regeneration. Furthermore, a recent report has demonstrated increased adipogenesis and a reduced number of Pax7+ satellite cells, perhaps dysfunctional, in regions of greater ischemia in human CLTI muscles.^57^ The persistent imbalance of pro- and anti-inflammatory macrophages in skeletal muscle has also been associated with impaired satellite cell activation and differentiation.^70^ More recently, a single-cell transcriptome analysis by Southerland et al revealed a pro-inflammatory macrophage signature in CLTI muscle and premature differentiation of satellite cells as the key features of failed muscle regeneration in the ischemic limb.^71^ Thus, another potential mechanism of hUC-MSC-mediated muscle regeneration is via satellite cell and MSC interactions and in this regard, MSCs have previously been shown to enhance proliferation and reduce apoptosis of satellite cells.^26,72^

At the molecular level, PAD has been associated with differential gene and miRNA expression in calf muscle^12,13^ as well as in circulation.^14-16^ We have previously shown a (predicted) dysregulated miRNA signature in human CLTI muscle, which was then validated in an HLI mouse model at 7 days post-ischemia.^30^ Herein, we report a sustained dysregulation of miR-1, miR-133a, and miR-29b at 28 days post-ischemic injury, and an upregulation of these miRNA levels after hUC-MSC delivery, with some inter-animal variability. Among the 3 miRNAs analyzed, miR-133a had the strongest effect and was the only statistically significantly upregulated miRNA after cell therapy (Figure 6b). However, there was a noticeable trend toward upregulation of miR-1 and miR-29b levels in hUC-MSC-treated mice, although the levels were more variable between animals. In addition, higher levels of miR-1, miR-133a, and miR-29b strongly correlated with increased myofiber size and muscle mass, and an overall decrease in ischemia-induced muscle damage (cISS). Cardiac and skeletal muscle-specific miR-1 and miR-133a are considered canonical “myomiRs” which play an essential role in regulating myogenesis and muscle development,^73-76^ while miR-29b is well known for its anti-fibrotic properties.^77-79^ These observations indicate the potential use of these miRNAs as biomarkers of muscle damage and/or their mechanistic role in skeletal muscle ischemia-induced changes.

Our previous analyses identified several extracellular matrix components targeted by these miRNAs which were upregulated 7 days after ischemia.^30^ Similarly, in this study, we observed sustained upregulation of pro-fibrotic markers at 28 days post-ischemia, including *Col1a1*, *Fn1*, and *Acta2*, and downregulation of these genes after hUC-MSC treatment. While *Col1a1* is a well-known target of miR-29b,^77,79^ and it has recently been shown to be a direct target of miR-133a,^80,81^*Fn1* has been shown to be a target of miR-1.^82^*Acta2* was also included in the analysis as it is known to be pro-fibrotic,^38^ although it is not a known target of these miRNAs, and its downregulation was not significant (*P = *.135). Recent studies have revealed the role of miR-1, miR-133a, and/or miR-29b in the regulation of fibrosis in several diseases including pulmonary fibrosis (miR-29b^79^) fibroplasia in the skin (miR-29b^83^), myocardial fibrosis (miR-133a^80^ and miR-1^84^) and arterial fibrosis (miR-1^85^). Here, we hypothesized the potential role of hUC-MSCs in regulating fibrosis in ischemic skeletal muscle by direct or indirectly modulating the levels of miR-1, miR-133a, and miR-29b in the muscle niche, as well as the expression of pro-fibrotic gene targets such as *Col1a1* and *Fn1*.

This study has some limitations. Although we examined the therapeutic effects of hUC-MSCs on skeletal muscle regeneration and repair after sustained ischemia (28 days), we did not explore the exact regulatory mechanisms by which hUC-MSCs exhibit such pleiotropic effects (Figure 7). Future research should examine the role of hUC-MSCs in regulating the cellular players in the ischemic muscle niche at earlier time points to further understand how MSCs may facilitate the appropriate environment resulting in enhanced regeneration processes. Secondly, further functional experiments are needed to elucidate whether the increase in miRNA levels is a consequence of the observed skeletal muscle repair, therefore, these miRNAs play a mechanistic role directly or indirectly mediated by hUC-MSC delivery, and/or whether these miRNAs may play a role as biomarkers of skeletal muscle damage. Third, mRNA target expression analysis using RNA isolated from FFPE skeletal muscle samples posed challenges. As suggested by Ma et al in,^36^ the final product may contain intact microRNAs and small RNAs; however, most mRNA and long RNA may be fragmented during the process of formalin fixation. While we, and others,^35^ had no limitations when detecting microRNAs using RT-qPCR from FFPE samples, we had technical limitations when detecting mRNAs, especially those lowly expressed. Finally, there is a limitation on the generalizability of our results to the CLTI human population, as only male, young, and healthy mice were used in this study.

## Conclusion

Herein, we report an hUC-MSC-mediated amelioration of skeletal muscle atrophy and function after ischemic damage via enhancement of myofiber regeneration, reduction of intermuscular adipocyte accumulation, tissue inflammation, and tissue fibrosis associated with the upregulation of miR-1, miR-133a, and miR-29b levels and downregulation of pro-fibrotic gene targets. Our results support the use of hUC-MSC therapy to ameliorate ischemia-induced skeletal muscle damage including muscle fibrosis in patients with CLTI, but also at earlier stages of the disease where skeletal muscle changes are also present. This approach may prevent disease progression and improve patients’ QOL. In addition, we hypothesized a potential role for these miRNAs as biomarkers of muscle damage and/or a mechanistic role in skeletal muscle ischemia-induced changes. This may open new avenues for manipulating miRNA expression as a potential therapeutic approach to restore skeletal muscle mass and function.

## Supplementary material

Supplementary material is available at *Stem Cells* online.

sxae058_suppl_Supplementary_Material

## Data Availability

The authors declare that the data supporting the findings of this study are available within the article and its supplementary information files and also available from the corresponding author upon request.
