# Supplementary material for: Mesenchymal stromal cell transplantation ameliorates fibrosis and microRNA dysregulation in skeletal muscle ischemia
Source: Stem Cells. 2024 Sep 16;42(11):976–91. doi: 10.1093/stmcls/sxae058 (PMC11541228; doi:10.1093/stmcls/sxae058)
Supplement: sxae058_suppl_Supplementary_Material [file sxae058_suppl_supplementary_material.pdf]

SUPPLEMENTARY MATERIAL.

Supplementary Material S1. Ambulatory score (walking ability) of left limb in mice in mice with hindlimb ischaemia surgery.

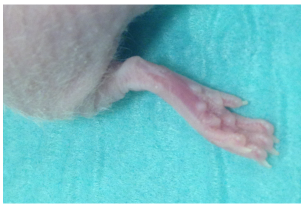

3 = Dragging the foot.

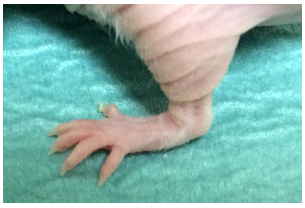

2 = No dragging the foot but no plantar flexion

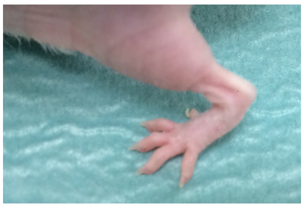

1 = Plantar flexion but no flexion of toes

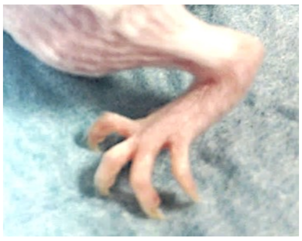

0 = Flexion of toes to resist traction of the tail similar to the non-operated foot.

<u>Supplementary Material S2.</u> Protocol for RNA extraction from FFPE skeletal muscle samples.

Ten tissue sections of 20μm thickness were cut from each paraffin block and placed in an RNase-free tube. Paraffin was melted at 55°C for 20 minutes. Sections were deparaffinised in 1mL of 100% Xylene at 55°C, two times, which was followed by two 1mL 100% per cent ethanol washes. The sample was then air-dried for approximately 10 minutes. 250uL of proteinase K digestion solution (Tris-HCl - 20mM pH 8.0, CaCl2 – 1mM, SDS – 0.5%, Proteinase K - 500uG/mL) was added and samples were incubated at 55°C for 3 hours and then 80°C for 15 minutes. RNA was isolated using 750μL TRIzol LS (Invitrogen). Samples were stored at -20ºC until further processing. Samples were thawed, vortex briefly transferred to a PhaseMaker™ tube (Invitrogen) and incubated for 5 minutes at room temperature. 200 μL of chloroform isoamyl alcohol mixture 24:1 (Supelco) was added and after 2 minutes of incubation, they were centrifuged at (15,000g, 4°C, 15 minutes). The aqueous phase was transferred to a new tube and 500 μL of isopropanol was added and then incubated at room temperature for 30 minutes. RNA was pelleted by centrifuging (15,000g, 4°C, 15 minutes) and the supernatant was discarded. The RNA was washed with 1mL 75% ethanol by vortexing and centrifuging (7,500g, 4°C, 5 minutes) and discarding the supernatant. The ethanol wash was repeated. After the removal of the ethanol supernatant, the tubes were centrifuged again for approximately 1 minute and any remaining supernatant was removed. RNA pellets were air dried for approximately 5 minutes. RNA was resuspended in 20μL RNase-free H2O, vortexed and spun briefly. RNA was solubilised by heating at 55°C for 10 minutes and samples were then placed on ice. 2uL of sample was assessed on a NanoDrop 2000 to determine RNA concentration and purity. 1μL of RNA was measured on a Qubit 4

fluorometer to assess RNA concentration. Concentrations as measured using the Qubit 4 were used for downstream calculations.

Limitations: As suggested by Ma et al in [36], the final product may contain intact microRNAs and small RNAs, however most mRNA and long RNA may be fragmented during the process of formalin fixation. While we, and others [35], had no limitations when detecting microRNAs using RT-qPCR, we had technical limitations when detecting mRNAs, especially lowly expressed mRNAs. In addition, in order to increase the chance of detecting mRNAs is it highly recommended to use primers with low amplicon size.

<u>Supplementary Material S3.</u> Table with mRNA primer sequences used in this study.

Primer Name	NCBI Gene ID	Sequence (Forward and Reverse)	PrimerBank ID
Rpl13a	22121	F 5' – AGCCTACCAGAAAGTTTGCTTAC<br>R 5' – GCTTCTTCTTCCGATAGTGCATC	334688867c2
Col1a1	12842	F 5' – GCTCCTCTTAGGGGCCACT<br>R 5' – CCACGTCTCACCATTGGGG	34328108a1
Fn1	14268	F 5' – GCTCAGCAAATCGTGCAGC<br>R 5' – CTAGGTAGGTCCGTTCCCACT	26344255a1
Acta2	11475	F 5' – GTCCCAGACATCAGGGAGTAA<br>R 5' – TCGGATACTTCAGCGTCAGGA	6671507a1

<u>Supplementary Material S4.</u> miRNA primers used in this study.

Target	Qiagen Catalog Number
Snord68	YP00203911
hsa-miR-1-3p	YP00204344
hsa-miR-133a-3p	YP00204788
hsa-miR-29b-3p	YP00204679

Supplementary Material S5. miRNA – mRNA target interaction analysis.

A) Validated miRNA-mRNA target interactions from miRTarBase.

	miRTarBase ID	
	**Mouse**	**Human**
miR-1(a)-3p: FN1	MIRT005017	MIRT006132
miR-133a-3p:COL1A1	-	MIRT021714
miR-29b-3p:COL1A1	MIRT000100	MIRT003678

B) Bioinformatic prediction by TargetScan.

<table>
<tr><th colspan="2">miR-1(a): Fn1</th></tr>
<tr><td>Position 1249-1256 of FN1 3' UTR
hsa-miR-1-3p</td><td>5' ...UUUAAUAAAAGAUUUACAUUCCA...
|||||||
3' UAUGUAUGAAGAAAUGUAAGGU</td></tr>
<tr><td>Position 1251-1258 of FN1 3' UTR
mmu-miR-1a-3p</td><td>5' ...UUAAUAAAAAGAUUUACAUUCCA...
|||||||
3' UAUGUAUGAAGAAAUGUAAGGU</td></tr>
<tr><th colspan="2">miR-133a: Col1a1</th></tr>
<tr><td>Position 194-200 of COL1A1 3' UTR
hsa-miR-133a-3p.2</td><td>5' ...UGACCAACCGAACAUGACCAAAA...
||||||
3' GUCGACCAACUUCCCCUGGUUU</td></tr>
<tr><td>Position 197-203 of COL1A1 3' UTR
mmu-miR-133a-3p.2</td><td>5' ...UGACCAACUGAACGUGACCAAAA...
|||| ||||||
3' GUCGACCAACUUCCCCUGGUUU</td></tr>
<tr><th colspan="2">miR-29b:Col1a1</th></tr>
<tr><td>Position 881-887 of COL1A1 3' UTR
hsa-miR-29b-3p</td><td>5' ...CCAUUUUAUACCAAAGGUGCUAC...
|||| ||||||
3' UUGUGACUAAAGUUUA----CCACGAU</td></tr>
<tr><td>Position 923-930 of COL1A1 3' UTR
hsa-miR-29b-3p</td><td>5' ...UGGGGAGGGAAUCACUGGUGCUA...
|||||||
3' UUGUGACUAAAGUUUACCACGAU</td></tr>
<tr><td>Position 1056-1062 of COL1A1 3' UTR
hsa-miR-29b-3p</td><td>5' ...GUGAAUUUUUCUAAAGGUGCUAU...
|||| ||||||
3' UUGUGACUAAAGUUUA--CCACGAU</td></tr>
<tr><td>Position 877-883 of COL1A1 3' UTR
mmu-miR-29b-3p</td><td>5' ...CAGUUUGGUAUCAAA-GGUGCUAC...
||||| ||||||
3' UUGUGACUAAAGUUUACCACGAU</td></tr>
<tr><td>Position 919-926 of COL1A1 3' UTR
mmu-miR-29b-3p</td><td>5' ...GUGGGAAGGAAUUUC---UGGUGCUA...
||||| |||||||
3' UUGUGACUAAAGUUUACCACGAU</td></tr>
<tr><td>Position 1095-1101 of COL1A1 3' UTR
mmu-miR-29b-3p</td><td>5' ...GUUUUUUUUCCUGAAGGUGCUAU...
|||| ||||||
3' UUGUGACUAAAGUUUA-CCACGAU</td></tr>
</table>
